# A3CarScene: An audio-visual dataset for driving scene understanding

**DOI:** 10.1016/j.dib.2023.109146

**Published:** 2023-04-12

**Authors:** Michela Cantarini, Leonardo Gabrielli, Adriano Mancini, Stefano Squartini, Roberto Longo

**Affiliations:** a Department of Information Engineering, Università Politecnica delle Marche, via Brecce Bianche 12, 60131 Ancona, Italy; b Groupe Signal Image et Instrumentation (GSII), École Supérieure d’Électronique de l'Ouest (ESEO), 10 Bd Jeanneteau, 49107 Angers, France; c Laboratoire d'Acoustique de l'Université du Mans (LAUM), UMR 6613, Institut d'Acoustique - Graduate School (IA-GS), CNRS, Le Mans Université, Av. Olivier Messiaen, 72085 Le Mans, France

**Keywords:** Acoustic and visual scene classification, Audio signal processing, Computer vision, Advanced driver assistance systems, Autonomous vehicles, Artificial neural networks, Multimodal deep learning

## Abstract

Accurate perception and awareness of the environment surrounding the automobile is a challenge in automotive research. This article presents *A3CarScene*, a dataset recorded while driving a research vehicle equipped with audio and video sensors on public roads in the Marche Region, Italy. The sensor suite includes eight microphones installed inside and outside the passenger compartment and two dashcams mounted on the front and rear windows. Approximately 31 h of data for each device were collected during October and November 2022 by driving about 1500 km along diverse roads and landscapes, in variable weather conditions, in daytime and nighttime hours. All key information for the scene understanding process of automated vehicles has been accurately annotated. For each route, annotations with beginning and end timestamps report the type of road traveled (*motorway, trunk, primary, secondar*y, *tertiary, residential*, and *service* roads), the degree of urbanization of the area (*city, town, suburban area, village, exurban* and *rural areas*), the weather conditions (*clear, cloudy, overcast*, and *rainy*), the level of lighting (*daytime, evening, night*, and *tunnel*), the type (*asphalt* or *cobblestones*) and moisture status (*dry* or *wet*) of the road pavement, and the state of the windows (*open* or *closed*).

This large-scale dataset is valuable for developing new driving assistance technologies based on audio or video data alone or in a multimodal manner and for improving the performance of systems currently in use. The data acquisition process with sensors in multiple locations allows for the assessment of the best installation placement concerning the task. Deep learning engineers can use this dataset to build new baselines, as a comparative benchmark, and to extend existing databases for autonomous driving.

**Specifications Table**

**Table:** 

Subject:	Signal Processing Computer Vision and Pattern Recognition Multimedia
Specific subject area:	Acoustic, visual, and multimodal scene understanding with deep learning algorithms for advanced driver assistance systems (ADAS).
Type of data:	Audio (*.wav) Video (*.mp4) Text files (*.csv)
How the data were acquired:	Data were acquired with a sensor-equipped vehicle while driving on public roads in the Marche Region, Italy.Car model: Mercedes A-ClassAudio equipment: • Behringer ECM8000 condenser microphones (no. 8) [1] • Roland Octa-Capture audio interface [2] • Notebook Apple MacBook Pro-A1286 • Audacity 3.2.4 multi-track audio editor and recorder [3] Video equipment: Mi DashCam 1S (no. 2) [4]
Data format:	Raw sensor data were stored in two forms. Microphone data were saved as wav audio and camera data as mp4 video. Annotations with corresponding timestamps associated with both data types were reported in text files in csv format. The dataset was organized into dedicated folders containing the recordings of each acquisition day.
Description of data collection:	Data were collected by driving planned routes with the sensor-equipped car and acquiring real-time audio and video data. Before departure, a check of the operation of all devices was carried out by initiating test recordings. The audio recordings were activated by turning on the audio interface and starting 8-channel recording using the *Audacity* software installed on the onboard laptop. Video recordings started automatically with the connection to the power supply.
Data source location:	City/Town/Region: Marche RegionCountry: ItalyLatitude and longitude for collected data: - Latitude from 43° 9′ 36″ to 43° 55′ 12″ N - Longitude from 12° 57′ 0″ to 13° 48′ 0″ E
Data accessibility:	Repository name: Open Science Framework (OSF) Data identification number: DOI 10.17605/OSF.IO/AJXU6 Direct URL to data: https://osf.io/ajxu6/

## Value of the Data

• This dataset includes more than 31 h of audio and video data recorded with eight microphones installed inside and outside the passenger compartment and two dashcams mounted on the front and rear windows of a research car. A wide range of driving scenarios is shown with diverse road infrastructure, urbanization contexts, weather and lighting conditions, and road pavement types and wetness. All this information is annotated and timestamped.
• This driving dataset can be a valuable resource for anyone involved in developing and testing advanced driving assistance systems and for automotive research in general. Acoustic and visual signals acquired with sensors in several setups aid in the technical evaluation and design of intelligent systems.
• Researchers and developers can leverage this real-world dataset to train and test deep learning algorithms for driving scene recognition using audio, video, or multimodal data. The dataset can also be useful for manufacturers to compare the effectiveness of their systems against those of their competitors.
• Several neural architectures can be employed for dataset analysis depending on the scope, such as extracting acoustic and visual features with Convolutional Neural Networks, capturing temporal dynamics with Recurrent Neural Networks, or generating new data from the existing ones with Generative Adversarial Networks.
• The dataset can be used for other applications, including the recognition of road damages, intersections, and audio warning signals, as well as the detection of obstacles out of sight. The object detection task can be accomplished by applying bounding boxes to elements of interest, such as vehicles, bicyclists, and pedestrians.
• Among existing publicly available solutions, this dataset of real-world driving scenarios is unique in the field, providing features not covered by other datasets for a complete understanding of the car's surroundings.

## 1 Objective

The context behind the generation of the dataset is related to audio research in the automotive field. While there is a great deal of related work in the computer vision area [5], datasets on machine listening focus mainly on sound event detection [6,7] and acoustic scene classification [8,9] in urban environments. Also, few works exist on audio-visual classification, e.g., involving dynamic environments [10], urban scenes [11], and urban traffic data [12]. Visual-acoustic multimodal data have been collected with an instrumented car in [13] to improve driving pleasantness by monitoring the state of the vehicle interior and in [14] for obstacle detection and tracking under vehicle vertical dynamics excitation caused by road anomalies. To the best of our knowledge, multisensor and multimodal recordings conducted in real-world scenarios with significant duration and consistent audio and video quality aimed at a complete comprehension of the car's surroundings are not available on public databases. The purpose of creating the dataset is to compare the performance of neural models trained in single- and multi-modality for developing intelligent systems to be installed in vehicles.

## 2 Data Description

*A3CarScene*[15] is an audio-visual dataset comprising more than 31 h of audio and video data recorded while driving a research car on public roads. The sensor equipment consists of eight microphones installed inside and outside the passenger compartment and two dashcams mounted on the front and rear windows of the vehicle.

**Fig. 1:**
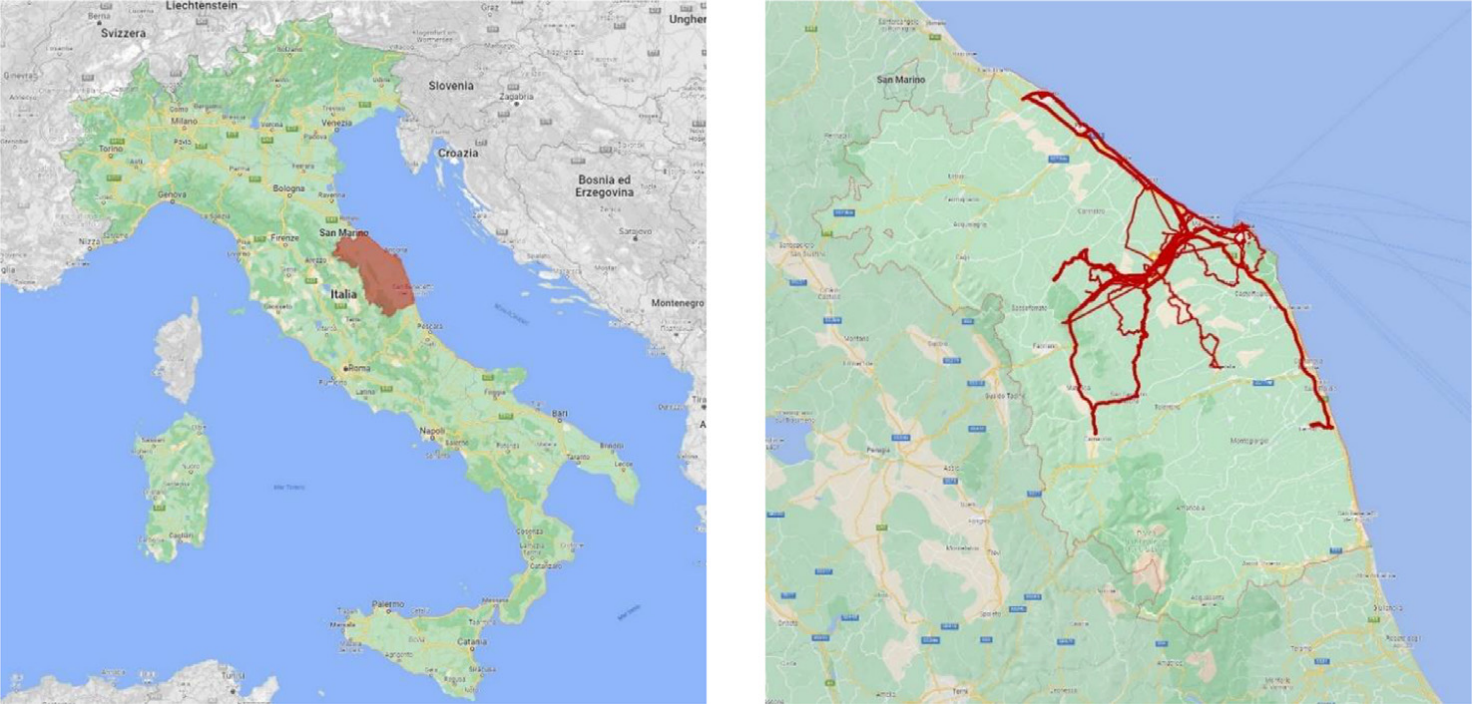
Location of the Marche Region in Italy (on the left) and routes traveled with the equipped car (on the right).

Acquisitions were made in the Marche Region, located in the center of Italy and characterized by variegated landscapes, from the coast in the east to the hilly areas in the center and the Apennine mountains in the west. Regarding its urbanization, the Marche Region presents two main urban centers (Pesaro and Ancona) and many towns with their respective suburban belts, exurban areas with industrial sites and infrastructure connections, and rural lands with scattered villages. The recording campaign was carried out in October and November 2022 for 14 days, covering different routes for a total of 1500 km. The itineraries were planned to encompass diverse areas, focusing on the central part of the region due to logistical reasons. Fig. 1 shows the location of the Marche Region and the routes traveled during the recording campaign.

The dataset consists of 400 files (320 audio and 80 video recordings). The files are organized into 14 folders, named with the acquisition date *yyyymmdd*, and each folder contains all audio and video files recorded on the same day. The duration of the files is variable, depending on the length of the itinerary or the cuts applied to individual recordings. The synchronized audio and video files inside each *yyyymmdd* folder are named with the criterion *file_type-device-yyyymmdd-part*.

**Fig. 2:**
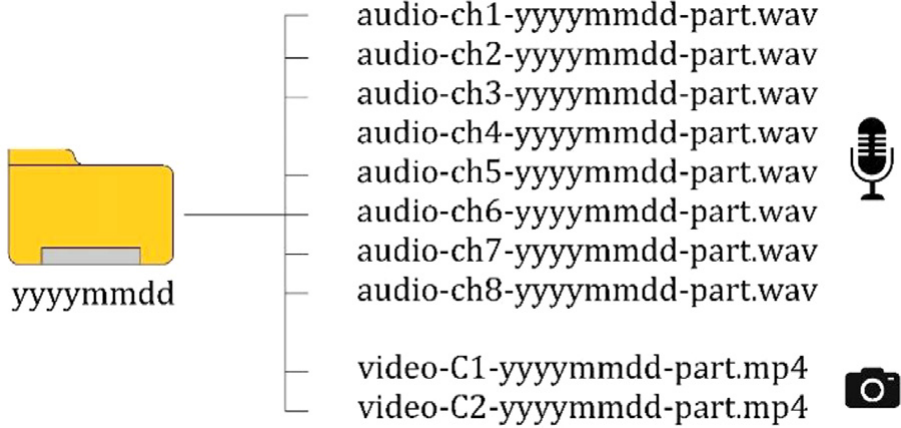
Organization of audio and video files inside a folder.

Audio recordings were stored in 8-channel tracks and exported separately, so audio-type files report the channel number (*ch1—ch8*) of the corresponding microphone as the device. Videos were shot with two cameras, where *C1* is the frontal and *C2* is the rear video device. Fig. 2 shows the generic contents of a folder.

Annotations are consistent for audio and video files and are reported in text files in csv format. The *metadata* folder contains the annotations of each recording date (*metadata-yyyymmdd.csv*) plus an overall one (*metadata.csv*), for a total of 15 csv files.

Table 1 shows the structure of metadata files reporting the name of the file (*filename*), timestamps (*start_time* and *end_time*), area identification number (*id*), and labeling categories (*road_type, deg_urb, weather, light, pav_type, pav_wetness*, and *window*). Each labeling category lists the corresponding attributes (or classes) that have been assigned.

**Table 1:** Structure of annotation files (*.csv).

filename	start_time	end_time	id	road_type	deg_urb	weather	light	pav_type	pav_wetness	window
yyyymmdd-part	hh:mm:ss	hh:mm:ss	No.	motorway trunk primary secondary tertiary residential service	city town suburban_area village exurban_area rural_area	clear cloudy overcast rainy	daytime evening night tunnel	asphalt cobblestones	dry wet	open closed

**Fig. 3:**
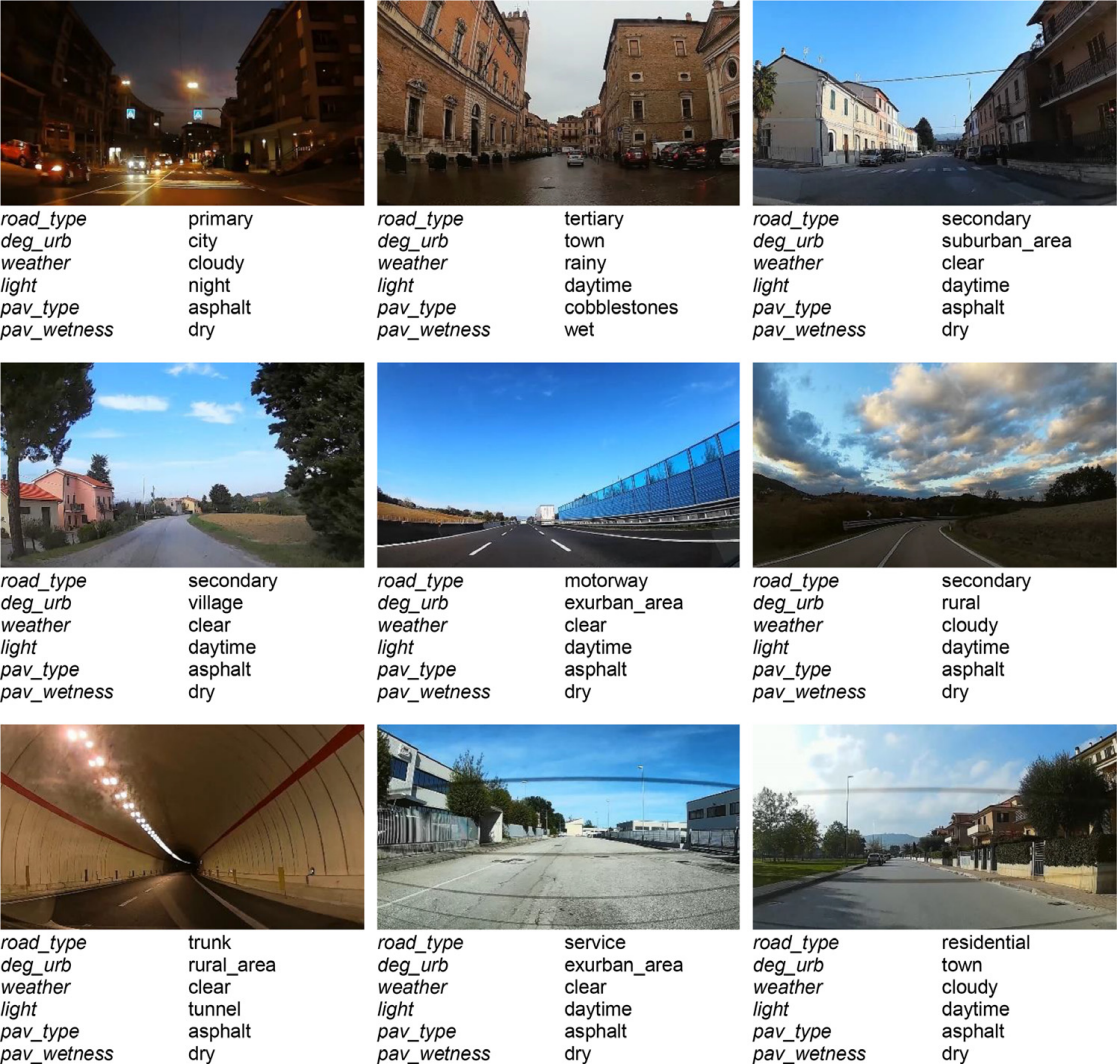
Video frames of the *A3CarScene* dataset with some associated annotations.

Fig. 3 shows examples of video frames of the routes with some associated annotations.

The individual columns of the annotation files are explained in detail as follows.

**Fig. 4:**
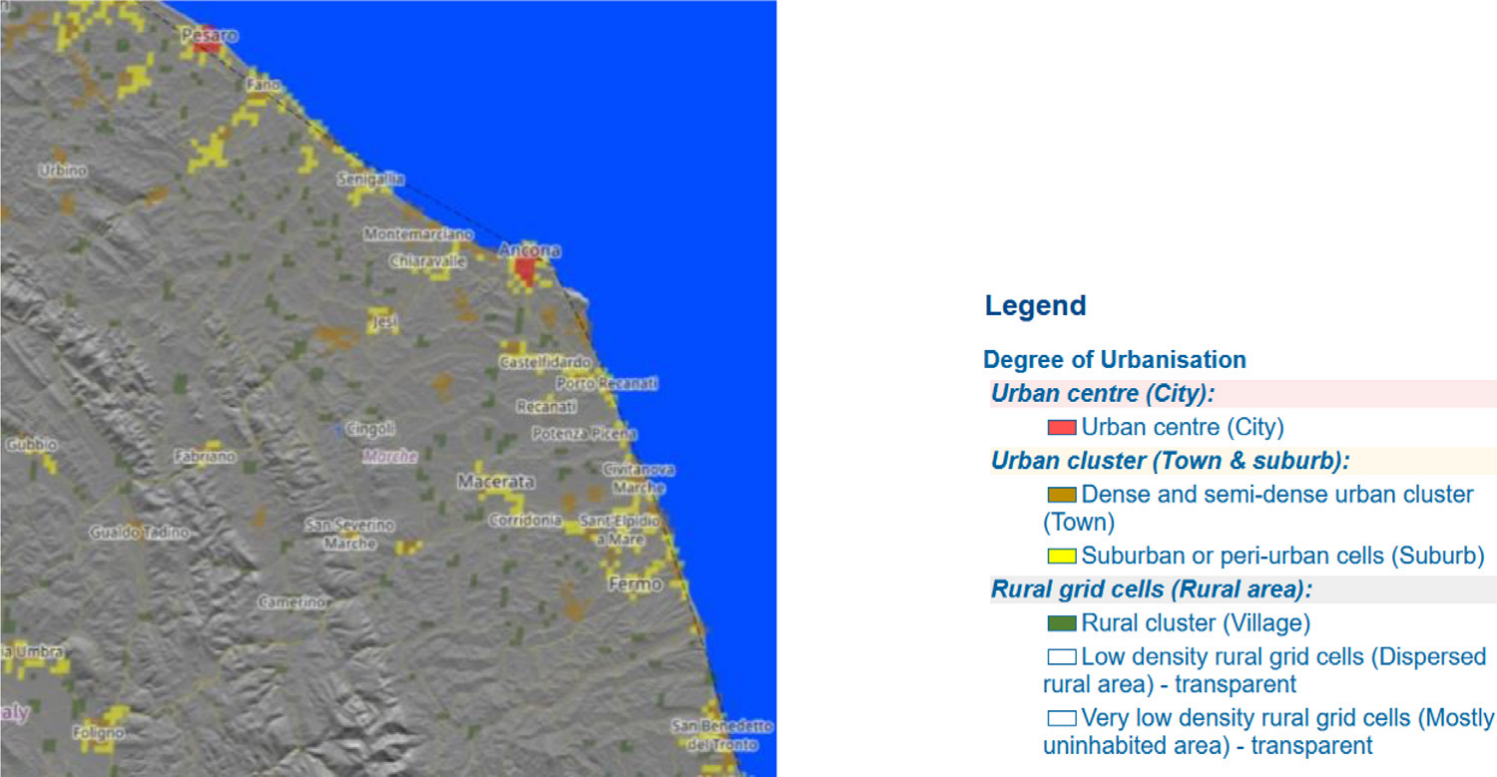
*Degree of urbanization* map of the Marche Region in Italy.
• *weather* describes the weather conditions detected during the route. The options are:
  1. Clear: sunny sky with no or insignificant cloud cover.
  2. Cloudy: sky with clouds but not completely covered.
  3. Overcast: sky completely covered with clouds.
  4. Rainy: overcast sky with light to moderate to significant rainfall.
• *light* relates to the time of day when recordings were made and passage in closed environments with artificial lighting (tunnels, covered parking lots). The following lighting conditions occur in the recordings: daytime, evening, night, and tunnel.
• *pav_type* represents the type of road pavement encountered in the routes, i.e., asphalt or cobblestones.
• *pav_wetness* indicates the moisture of the road pavement (dry or wet).
• *window* indicates the state of the windows during the recordings (open or closed). This feature is not descriptive of the car's surroundings but was included to assess the impact of the external noise on the performance of the audio sensors inside the passenger compartment.

• *filename* is the string common to audio and video filenames, expressed by the date and the part of the recording belonging to the same day (*yyyymmdd-part*).
• *start_time* and *end_time* are the timestamps in which the scene has uniform labeling, expressed in *hh:mm:ss* format.
• *id* represents the number that identifies the area covered, ranging from 001 to 407. The *id* can indicate a single road section or a group of neighboring roads belonging to the same type of infrastructure and degree of urbanization. The purpose of the *id* assignment is related to the split of the dataset into training and test sets so that routes different from those used in the training phase can be chosen for inference.
• *road_type* represents the road classification typology according to *OpenStreetMap* (OSM) [16], the free geographic database updated and maintained by a community of volunteers through open collaboration. The choice of this classification is related to the worldwide use of OSM and international equivalence between road infrastructure types. In the following, the description of the infrastructures traveled and the equivalence with the Italian regulations according to the Legislative Decree No. 285 of April 30, 1992 “Codice della Strada” (https://www.bosettiegatti.eu/info/norme/statali/1992_0285.htm, accessed 30 January 2023) are given.
  1. Motorway: limited access highway with tolls and interchanges (in Italy, A-category road).
  2. Trunk: ring road or expressway, also a road of minor importance having interchanges instead of grade-separated intersections (in Italy, B-category road).
  3. Primary: national, regional, or provincial road of major importance, e.g., that connecting provincial capitals and thus of national significance (in Italy, B-category road).
  4. Secondary: another regional or provincial road of minor importance (in Italy, C-category road).
  5. Tertiary: main urban road (in Italy, D-category road).
  6. Residential: road in an urban residential area (in Italy, E-category road).
  7. Service: a service way to access, for example, a non-residential area, a parking lot, or a private area (in Italy, F-category road).
• *deg_urb* represents the classification that indicates the character of an area. It is inspired by the *Degree of urbanization* [17], a methodology for the delineation of cities and urban and rural areas for international and regional statistical comparison purposes endorsed by the United Nations Statistical Commission. The *Degree of urbanization* classifies the entire territory of a country along the urban-rural continuum, combining population size and density thresholds to capture the full settlement hierarchy. Global Human Settlement data with global coverage, as illustrated in Fig. 4, can be viewed interactively on the https://ghsl.jrc.ec.europa.eu/visualization.php# website (accessed 30 January 2023). According to the *Degree of urbanization* legend, territories are represented by the following attributes.
  1. City: urban center.
  2. Town: dense and semi-dense urban cluster.
  3. Suburban area: suburban and peri‑urban cells.
  4. Village: rural cluster.
  5. Exurban area: low-density grid cells (industrial sites, semi-rural or mixed-use areas).
  6. Rural area: very low-density rural grid cells.

The dataset includes 31 h, 20 min and 8 s of recordings for each audio and video sensor. The individual classes in each labeling category are unbalanced proportionally to the territory characteristics and weather conditions encountered during the acquisition campaign.

Table 2 shows the amount of audio and video material for all classes associated with each labeling category, quantified according to recording duration in hh:mm:ss. Table 3 shows the length and classes present in the individual recordings contained in each folder.

**Table 2:** Audio and video material for all the labeling categories [hh:mm:ss].

road_type	duration [hh:mm:ss]	deg_urb	duration [hh:mm:ss]	weather	duration [hh:mm:ss]	light	duration [hh:mm:ss]	pav_type	duration [hh:mm:ss]	pav_wetness	duration [hh:mm:ss]	window	duration [hh:mm:ss]
motorway trunk primary secondary tertiary residential service	02:10:15 02:54:01 03:28:06 14:57:40 04:26:09 01:45:44 01:38:13	city town suburban_area village exurban_area rural_area	02:03:44 08:15:11 02:19:53 01:51:21 08:54:13 07:55:46	clear cloudy overcast rainy	13:02:49 07:15:42 07:46:21 03:15:16	daytime evening night tunnel	25:11:00 03:00:53 02:45:03 00:23:12	asphalt cobblestones	31:12:12 00:07:56	dry wet	26:19:37 05:00:31	open closed	27:39:12 03:40:56

**Table 3:** General features of the individual files that compose the dataset.

folder	filename	duration	road_type	deg_urb	weather	light	pav_type	pav_wetness	window

20221011	20221011-1	00:55:38	secondary, tertiary, residential, service	town, suburban_area, exurban_area	overcast	daytime	asphalt, cobblestones	dry	open, closed
20221014	20221014-1	00:25:31	primary, secondary, tertiary, residential, service	town, exurban_area	clear, cloudy	daytime	asphalt	dry	open
	20221014-2	00:56:05	primary, tertiary, residential, service	town, suburban_area, exurban_area	clear	daytime	asphalt	dry	open
	20221014-3	00:39:24	primary, secondary, tertiary, service	town, suburban_area, village, exurban_area, rural_area	clear, cloudy	daytime	asphalt	dry	open
20221017	20221017-1	00:35:33	secondary, residential, service	town, suburban_area, village, exurban_area, rural_area	clear, cloudy	daytime	asphalt	dry	open
	20221017-2	00:14:50	secondary	village, rural_area	clear	daytime	asphalt	dry	open
	20221017-3	00:11:29	trunk, secondary	rural_area	clear	daytime, tunnel	asphalt	dry	open, closed
	20221017-4	00:37:59	trunk, secondary, residential	town, suburban_area, exurban_area, rural_area	clear	daytime, tunnel	asphalt	dry	open
	20221017-5	00:53:59	secondary, tertiary, residential, service	town, suburban_area, exurban_area	clear	evening, night	asphalt	dry	open
20221018	20221018-1	00:41:03	trunk, primary, secondary, tertiary, service	town, suburban_area, exurban_area	clear	daytime, tunnel	asphalt	dry	open
	20221018-2	00:21:00	secondary, tertiary, service	town, exurban_area, rural_area	clear	daytime, tunnel	asphalt	dry	open
	20221018-3	01:10:57	trunk, primary, secondary, tertiary, residential	city, town, suburban_area, exurban_area, rural_area	clear	daytime, evening, tunnel	asphalt	dry	open
20221019	20221019-1	01:01:17	primary, secondary, tertiary, residential	town, suburban_area, exurban_area, rural_area	clear	daytime	asphalt	dry	open
	20221019-2	01:16:09	primary, secondary, tertiary, residential, service	town, suburban_area, village, exurban_area, rural_area	clear	daytime, evening	asphalt	dry	open
20221021	20221021-1	01:03:07	secondary, tertiary, residential	town, suburban_area, village, exurban_area, rural_area	overcast, rainy	daytime	asphalt	dry	open, closed
	20221021-2	00:38:02	secondary, residential, service	town, suburban_area, exurban_area, rural_area	overcast, rainy	daytime	asphalt, cobblestones	dry	open
20221024	20221024-1	00:47:30	trunk, primary, secondary, tertiary, residential, service	city, town, suburban_area, exurban_area	clear	daytime, tunnel	asphalt	dry	open, closed
	20221024-2	01:12:08	primary, secondary, tertiary, residential, service	city, town, suburban_area, exurban_area	clear, cloudy	night, tunnel	asphalt	dry	open
20221025	20221025-1	01:19:23	trunk, secondary, tertiary, service	town, village, exurban_area, rural_area	clear, cloudy	daytime	asphalt	dry	open
20221108	20221108-1	00:17:59	secondary, tertiary, residential, service	town, exurban_area	clear	daytime	asphalt	dry	open
	20221108-2	00:14:32	secondary	town, suburban_area, exurban_area	clear	daytime	asphalt	dry	open
	20221108-3	00:55:15	primary, secondary, tertiary, service	town, suburban_area, village, exurban_area, rural_area	clear	daytime, tunnel	asphalt	dry	open
	20221108-4	00:34:02	secondary, tertiary, residential, service	town, suburban_area, exurban_area	clear, cloudy	daytime	asphalt	dry	open
	20221108-5	01:22:10	secondary, tertiary, residential, service	town, suburban_area	cloudy	evening, night, tunnel	asphalt, cobblestones	dry	open
20221110	20221110-1	00:55:52	motorway, trunk, secondary, tertiary, residential	town, exurban_area	cloudy, overcast	daytime, tunnel	asphalt	dry	open, closed
	20221110-2	00:15:14	motorway, primary, secondary, tertiary, service	city, suburban_area, exurban_area	overcast	daytime	asphalt	dry	open
	20221110-3	01:30:52	primary, secondary, tertiary, service	city, town, suburban_area, village, exurban_area, rural_area	overcast	daytime	asphalt	dry	open
20221115	20221115-1	01:28:20	trunk, primary, secondary, tertiary, service	city, town, suburban_area, village, exurban_area, rural_area	rainy	daytime, tunnel	asphalt	wet	closed
	20221115-2	00:35:59	trunk, secondary, tertiary, service	suburban_area, exurban_area	rainy	evening, night, tunnel	asphalt	wet	closed
	20221115-3	00:17:29	secondary, tertiary, service	town, exurban_area	rainy	night	asphalt	wet	closed
20221116	20221116-1	01:07:33	trunk, primary, secondary, tertiary, service	town, suburban_area, village, exurban_area, rural_area	overcast, rainy	daytime, tunnel	asphalt	wet	open
	20221116-2	01:31:10	primary, secondary, tertiary, residential, service	town, suburban_area, village, exurban_area, rural_area	overcast, rainy	daytime, tunnel	asphalt	wet	open
20221117	20221117-1	01:13:59	motorway, trunk, primary, secondary, residential, service	town, suburban_area, exurban_area, rural_area	overcast	daytime, tunnel	asphalt	dry	open, closed
	20221117-2	01:04:29	motorway, trunk, primary, secondary, tertiary, service	town, suburban_area, exurban_area, rural_area	clear, cloudy, overcast	daytime, tunnel	asphalt	dry	open
	20221117-3	00:37:30	secondary, tertiary, residential, service	town, village, rural_area	cloudy	daytime	asphalt	dry	open
	20221117-4	00:26:59	secondary, tertiary, residential	village, rural_area	cloudy	daytime	asphalt, cobblestones	dry	open
	20221117-5	00:19:25	secondary, residential	town, village, rural_area	cloudy	daytime, evening	asphalt	dry	open
20221124	20221124-1	00:23:41	secondary, tertiary, residential	city, town, suburban_area, exurban_area	clear	daytime	asphalt	dry	closed
	20221124-2	00:26:31	trunk, primary, secondary, tertiary, residential	city, suburban_area	clear	daytime, tunnel	asphalt	dry	open
	20221124-3	00:40:03	trunk, primary, secondary, tertiary, residential, service	city, town, suburban_area, exurban_area	clear	daytime, tunnel	asphalt	dry	open

## 3 Experimental Design, Materials and Methods

### 3.1 Acquisition Stage

A Mercedes A-Class research car model equipped with audio and video sensors was used for the recording campaign. The audio setup has already been employed in another research [18]. Recordings were made with only the driver or, at most, one passenger on board. The vehicle was driven within the speed limits imposed by the road infrastructure and with the windows either open or closed as desired. No music sources were activated during driving, and no dialog was present.

#### 3.1.1 Audio Setup

The audio equipment consisted of eight measurement condenser microphones model Behringer ECM8000 connected via XLR connectors to an 8-channel Roland Octa-Capture soundboard, which in turn was interfaced via USB to a laptop computer. The soundboard and laptop computer were connected to the power supply of the car via a DC/AC power inverter. The installation comprised four microphones inside the passenger compartment (two on either side of the front seats and two on either side of the rear seats at seatback height), two in the trunk, and two on opposite sides behind the license plate. The audio interface was installed in the trunk, and the laptop computer was placed on the rear seats. The recordings were made separately in eight channels, corresponding to the eight microphones, and the start/end times were managed using the open-source software *Audacity* installed on the laptop. A sampling rate of 44.1 kHz and 32-bit encoding was set for the audio files, and at the end of the recordings, the individual channel tracks were saved in wav format.

#### 3.1.2 Video Setup

The video equipment consisted of two cameras Mi DashCam 1S attached with the dedicated mount to the front and rear windows of the car. The front camera was secured to the right of the rearview mirror so as not to interfere with the driver's view, while the rear camera was placed in the high-center position of the rear window. The cameras were powered via a USB cable connected to the USB ports included in the car. Video data were captured with a 1920 × 1080 pixels resolution and variable fps for up to 30 fps and saved 2-minute segments in mp4 format. The two cameras are equipped with an internal clock that enables the synchronization of video data. Audio data recording is optional and has been enabled to facilitate synchronization with audio devices.

**Fig. 5:**
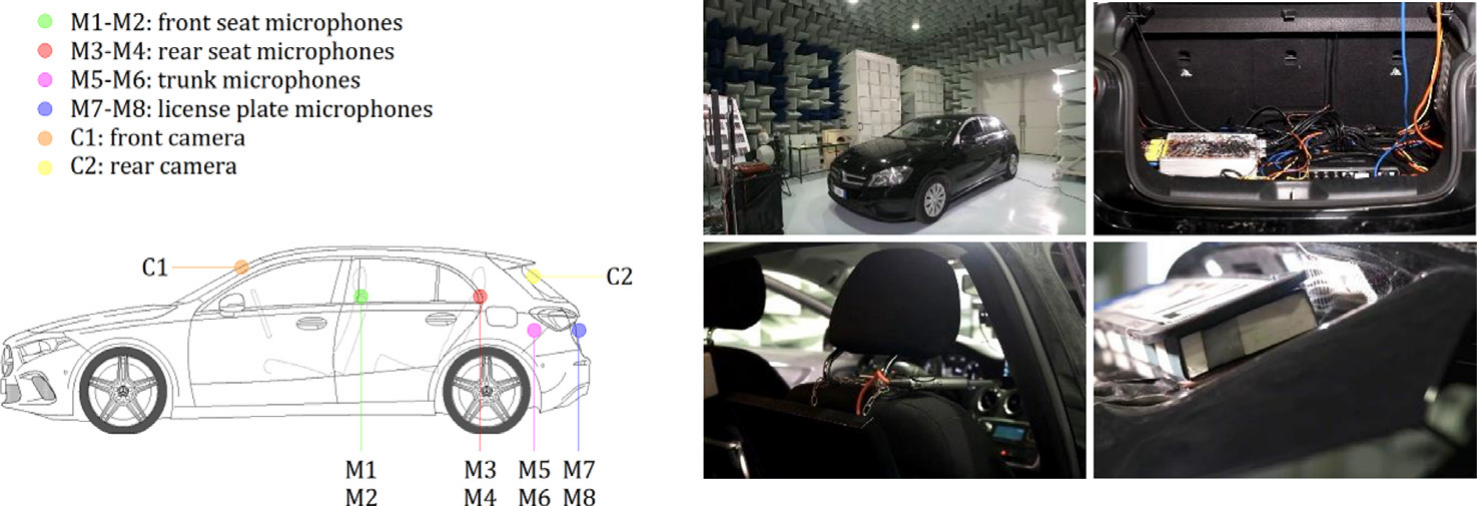
Overview of the sensor placement (on the left) and details of the equipment (on the right).

**Fig. 6:**
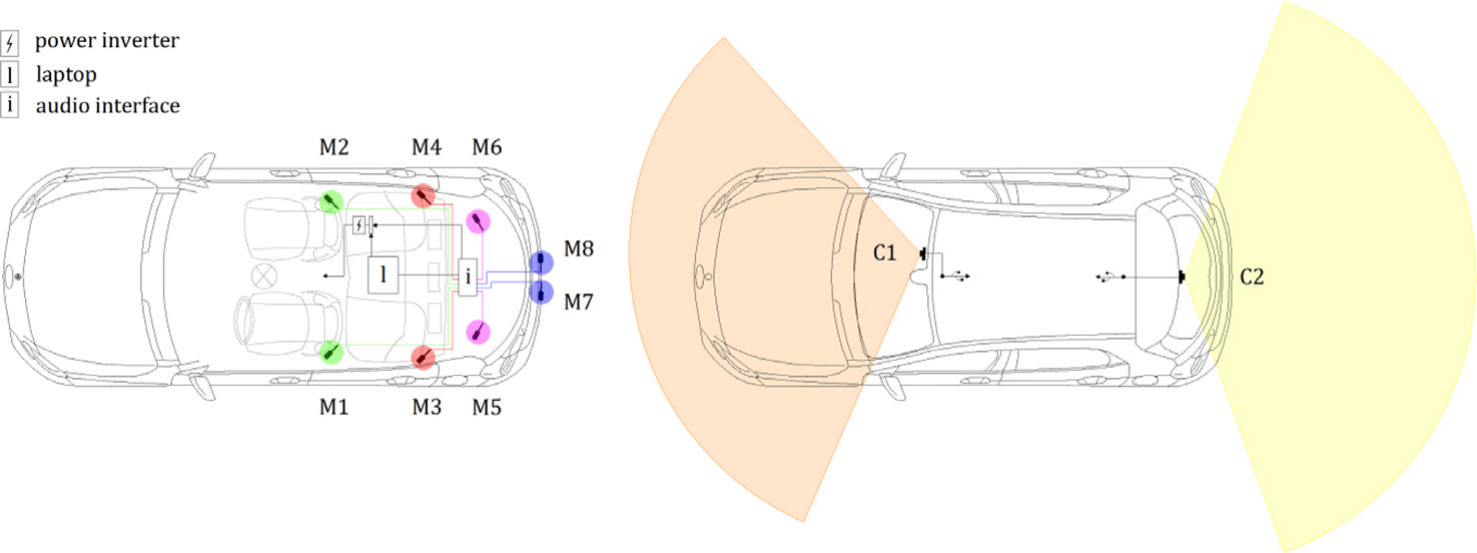
Setup of audio (on the left) and video (on the right) devices.

Table 4 lists the main technical specifications of audio and video recording devices. Fig. 5 shows an overview of sensor placement and some photographs with equipment details, and Fig. 6 schematizes the configuration of the audio and video devices.

**Table 4:** Main technical specifications of audio and video recording devices.

Device	Main Specifications
Behringer ECM8000 microphone	Type: elect. condenser. Polar Pattern: omnidirectional. Impedance: 200 Ohms. Sensitivity: 70 dB. Frequency Response: 20–20 000 Hz. Connector: gold-plated XLR. Phantom Power: +15 to +48 V. Weight: 136 g.
Roland Octa-Capture audio interface	Number of audio channels: 8. Nominal Input Level: input jack 1—6 (XLR type) −56 to −6 dBu, input jack 7—8 (XLR type) −50 to +0 dBu. Nominal Output Level: +0 dBu (balanced). Headroom: 16 dB. Input Impedance: input jack 1—6 (XLR type) 5 k ohms (balanced), input jack 7—8 (XLR type) 10 k ohms (balanced). Output Impedance: 1.8 k ohms (balanced). Frequency Response 44.1 kHz: 20 Hz to 20 kHz (+0/−2 dB). Power Supply: DC 9 V (AC adaptor). Current Draw: 1.45 A. Dimensions: 283.8 (W) x 157.9 (D) x 50.4 (H) mm. Weight: 1.32 kg
Mi DashCam 1S	Dimensions: 87.5 (W) x 18 (D) x 53 (H) mm. Input: 5 V, 1.5 A. Image Sensor: Sony IMX307. Resolution: 1080 p. Camera: FOV 140°, F1.8, 6-glass lens. Frame Rate: variable. Working Frequency: 2412—2472 MHz. Operating Temperature: −10 °C—60 °C.

### 3.2 Processing Stage

Processing operations were carried out to synchronize audio and video data and to apply data protection laws. Specifically, the following procedures were performed for each recording associated with a specific route.

• Video data from each camera, recorded in 2-minute segments, were merged into a single video file and exported in mp4 format at 25 fps. The synchronization of front and rear videos was verified by comparing the time-frequency representations of the audio acquired by each camera. Open-source software *kdenlive*, based on the *ffmpeg* library, and *Audacity*, were used to perform the processing operations on video and audio data, respectively.
• Using the audio tracks recorded by the cameras, video data were aligned with the 8-channel recordings from the microphones, which were then exported to separate tracks in wav format, keeping the 44.1 kHz sampling rate and 32-bit encoding unchanged.
• To comply with General Data Protection Regulation guidelines, license plates and faces were censored with the open-source python tool *DashcamCleaner* available at the url https://github.com/tfaehse/DashcamCleaner (accessed 30 January 2023). It is based on the YOLOv5 [19] algorithm for automatic license plate and face recognition using pre-trained models with different parameters that adjust training image resolutions, network depths, and dataloaders. Video files were blurred with *720p_medium_mosaic* option, kernel radius of the gaussian filter of 30, and the quality of the resulting video equal to 5.
• Lastly, video and audio data for each itinerary were played simultaneously in the *kdenlive* software for the manual labeling phase. For each class, markers were applied corresponding to the start and end of each homogeneous context in the road, urban, meteorological, and temporal domains and annotated in a csv file.

## Ethics statements

This work did not include work involved with human subjects, animal experiments or data collected from social media platforms.

## CRediT authorship contribution statement

**Michela Cantarini:** Conceptualization, Methodology, Software, Formal analysis, Investigation, Data curation, Writing – original draft. **Leonardo Gabrielli:** Conceptualization, Methodology, Formal analysis, Resources. **Adriano Mancini:** Conceptualization, Methodology, Formal analysis, Resources, Writing – review & editing. **Stefano Squartini:** Conceptualization, Writing – review & editing, Supervision, Project administration, Funding acquisition. **Roberto Longo:** Conceptualization, Investigation, Data curation, Writing – review & editing, Supervision.

## Declaration of Competing Interest

The authors declare that they have no known competing financial interests or personal relationships that could have appeared to influence the work reported in this paper.

## Data Availability

A3CarScene (Original data) (Open Science Framework (OSF)).
